# Differential health outcomes of the COVID‐19 pandemic among minority populations: An analysis based on Chicago's neighborhoods

**DOI:** 10.1002/puh2.111

**Published:** 2023-07-21

**Authors:** Simon Geletta, Kenneth Soyemi

**Affiliations:** ^1^ Department of Public Health Des Moines University Des Moines Iowa USA; ^2^ Cook County Health Systems Chicago Illinois USA

**Keywords:** COVID‐19 outcome disparities, health disparities, minority health, race‐ and ethnicity‐based health outcome patterns

## Abstract

**Background:**

The study examines the effects of the COVID‐19 pandemic on different ethnic and racial groups. It aims to investigate the existence or nonexistence of significant variations in COVID‐19 health outcomes among two ethnic and racial minorities that resided in Chicago neighborhoods during the onslaught of the pandemic. Researchers have traditionally studied health disparities by comparing the health of minorities representing “underserved” populations and those with adequate healthcare. This study focuses on the heterogeneity of health outcomes between different minority populations, mainly Black and Hispanic, traditionally considered underserved populations.

**Methods:**

This cross‐sectional study uses secondary data from a public reporting site. The unit of analysis is neighborhood units based on US postal zip codes that are cross‐referenced with the US Census Bureau's zip code tabulation area codes. We used Chicago neighborhood data and applied geographic analyses to describe the patterns of similarities and differences in the outcomes of the COVID‐19 pandemic among neighborhoods with different ethnic and racial minorities residing in them. Using the one‐way analysis of variance technique, we also tested research hypotheses about the COVID‐19 outcome differences and/or similarities among the neighborhoods.

**Results:**

Our findings show that although Hispanic neighborhoods disproportionately carried a higher burden of infection by the disease, the mortality due to the illness or the case fatality rate was not much higher than in the other neighborhoods. In contrast, African American neighborhoods did experience significantly higher case fatality rates—although their infection rate was not statistically significantly higher than the average infection rates of the other Chicago neighborhoods.

**Conclusions:**

Minority status creates distinct adverse effects on different minority groups. The patterns of distinct outcomes need to be well understood through further studied and considered by policymakers when health policies are designed to address the impact of health disparities.

## BACKGROUND

Since the early days of the COVID‐19 pandemic, there has been widespread recognition that minorities and socioeconomically disadvantaged individuals were disproportionately affected by COVID‐19. Several studies have established that Blacks and Hispanics are disproportionately impacted by the COVID‐19 pandemic in many communities around the United States [1, 2, 3, 4, 5, 6].

A 2020 study that analyzed COVID‐19 testing and mortality data from New York City found that Black and Hispanic individuals had higher COVID‐19 positivity rates and mortality rates compared to non‐Hispanic whites [6]. Similarly, in a 2022 study, Black and Hispanic individuals were likelier to report COVID‐19‐related symptoms, testing, and diagnoses compared to non‐Hispanic whites [7]. In a study that analyzed COVID‐19 testing and mortality data from Cook County, Illinois, Black, and Hispanic individuals had higher COVID‐19 positivity and mortality rates compared to non‐Hispanic Whites [8].

The findings of some of these studies linked this disproportionate effect with the disproportionate presence of chronic conditions, which represent underlying medical conditions that exacerbate the outcome of COVID‐19 infection; such conditions include hypertension, diabetes mellitus, HIV/AIDS, cancer, cardiovascular disease, and obesity, which disproportionately affect ethnic minorities and the poor in terms of higher incidence or worse outcomes [9, 10]. Accordingly, an observational study that used the 2019 behavioral risk factors surveillance system data directly estimated that people who are “black, American Indian, or live in low‐income households” are more likely to have conditions associated with an increased risk of illness from COVID‐19 relative to those who are White or are living in higher income households. It concluded that the inequities in risk are compounded by structural disparities in access to medical insurance, wealth, and income volatility [11].

Two central tenets run through the findings of the above‐reviewed literature. First, there is the implication that the spread of the COVID‐19 pandemic identically impacted all individuals who are members of the “underserved population,” including Blacks, Hispanics, low socioeconomic status individuals, and other similarly disadvantaged members of our society. The second is that the disproportionate effects in terms of both morbidity and mortality among these minority populations might not be limited to the existence or high probability of confounding factors among these populations and that it may be linked to their underserved or underprivileged status. These deductions are reasonable conclusions that need to be bolstered by sound evidence.

In terms of the research approaches that investigators used to study this topic, a few used individual‐level prevalence and/or incidence data. Many investigations based their observations on geographic or community‐level data [5, 6, 12–14]. The choice is driven by the difficulty of obtaining individual‐level incidence data and the ready availability of geographically reported public use data. The use of geographic or community‐level data to depict ethnic and racial health disparities is justified by the fact that settlement neighborhoods in most US cities are segregated by race, ethnicity, and economic factors. Accordingly, most studies that used a local area geographic approach to investigate disparities in COVID‐19 testing, infection, and deaths used large metropolitan communities as data sources for their studies. Although most of the studies focused on the city of New York, a few were based on Chicago or Cook County data. One particular study in Chicago used socioeconomic patterns to construct a “social vulnerability” index [15]. The study combined race, ethnicity, and other demographic and socioeconomic variables to create the vulnerability index. The study demonstrated a higher burden of illness due to COVID‐19 among the communities with a higher social vulnerability score than those with a higher social vulnerability score as compared to those with a lower social vulnerability score.

A study that used Cook County, IL data employed the same approach to the study of minorities and suggested that more than socioeconomic status, race is a significant factor for COVID‐19‐related mortality in Cook County [13]. This study was the first to create a context in which the differential outcomes of the COVID‐19 pandemic could be compared among different sections of disadvantaged populations. However, the study failed to address the differential impacts more comprehensively. It did report that Blacks were more likely to die from COVID‐19, and Hispanic individuals died at younger ages than Whites.

A summary report by the City of Chicago shows that over the first 6 months of the COVID‐19 pandemic, the prevalence and case fatality rate of the disease among the African American population was 29.6% and 44.4%, respectively [16]. The report detailed that the Hispanic population, which constitutes about the same proportion of the city of Chicago, experienced prevalence and case fatality rates of 47.7% and 31.1%, respectively. Non‐Hispanic Whites, which constituted about half of the city's population, had a prevalence rate of 14.4% and a case fatality rate of 18.9% [15]. This pattern indicates that the Hispanic population was most vulnerable in terms of prevalence, whereas the African American population was most vulnerable in terms of mortality. Non‐Hispanic Whites have lower prevalence and case fatality rates from COVID‐19 than the two race and ethnic categories.

When we examine the patterns 2 years later, we find that the COVID‐19 pandemic prevalence and case fatality rate among African Americans is 22.2% and 42.1%, respectively [16]. Among the Hispanic population, the prevalence and case fatality rates are 27.7% and 29.6%, respectively, and among the non‐Hispanic Whites, 25.7% and 23.1%, respectively. As observed, the current prevalence of COVID‐19 among Chicago residents is the lowest among African Americans, and the case fatality rate is the highest. Hispanics still carry the highest prevalence rate. Such patterns of distinctions warrant examining the details of the interplay between disease prevalence and the different ethnic, racial, and socioeconomic groups.

As our literature review reveals, most studies investigating health disparities have aimed to show the existence of inequalities in general. In recent times, such focus has resulted in the creation of the “index” of disadvantaged status by lumping together different demographic and socioeconomic characteristics of the population. Although such an approach is helpful in drawing attention to the correlation between disadvantaged status and poor health outcomes, it can also have the adverse effect of masking the precise relationships between the two.

Statistical reports and previous studies show the existence of general health disparities based on race/ethnicity and related socioeconomic conditions such as poverty and education. Thus, this study aims to examine how racial and ethnic differences parallel differential outcomes in terms of COVID‐19 morbidity and mortality. It provides evidence that supports that the pandemic's impact on “underserved populations” is not identical across the board. This study goes beyond describing the relationship between disadvantaged status and poor health outcomes—which are commonly labeled “health disparities,” “health inequities,” or “health inequalities” and analyzes the patterns of the inequities among different disadvantaged populations in terms of health outcomes. In this instance, our focus is on the plight of ethnic and racial groups.

## METHODS

### Study design, population, and setting

This is a cross‐sectional study based on neighborhood‐level data from the city of Chicago. Chicago is the third‐largest city in the United States and is located in Illinois, a state in the midwestern part of the country. As of the 2020 United States Census, Chicago had a population of approximately 2.7 million people of which 32.8% are White, 30.1% Black or African American, and 29% Hispanic or Latino (of any race). This racial and ethnic breakdown makes Chicago ideal for analyzing the impact of differential outcomes of COVID‐19 among the racial and ethnic groups.

**FIGURE 1:**
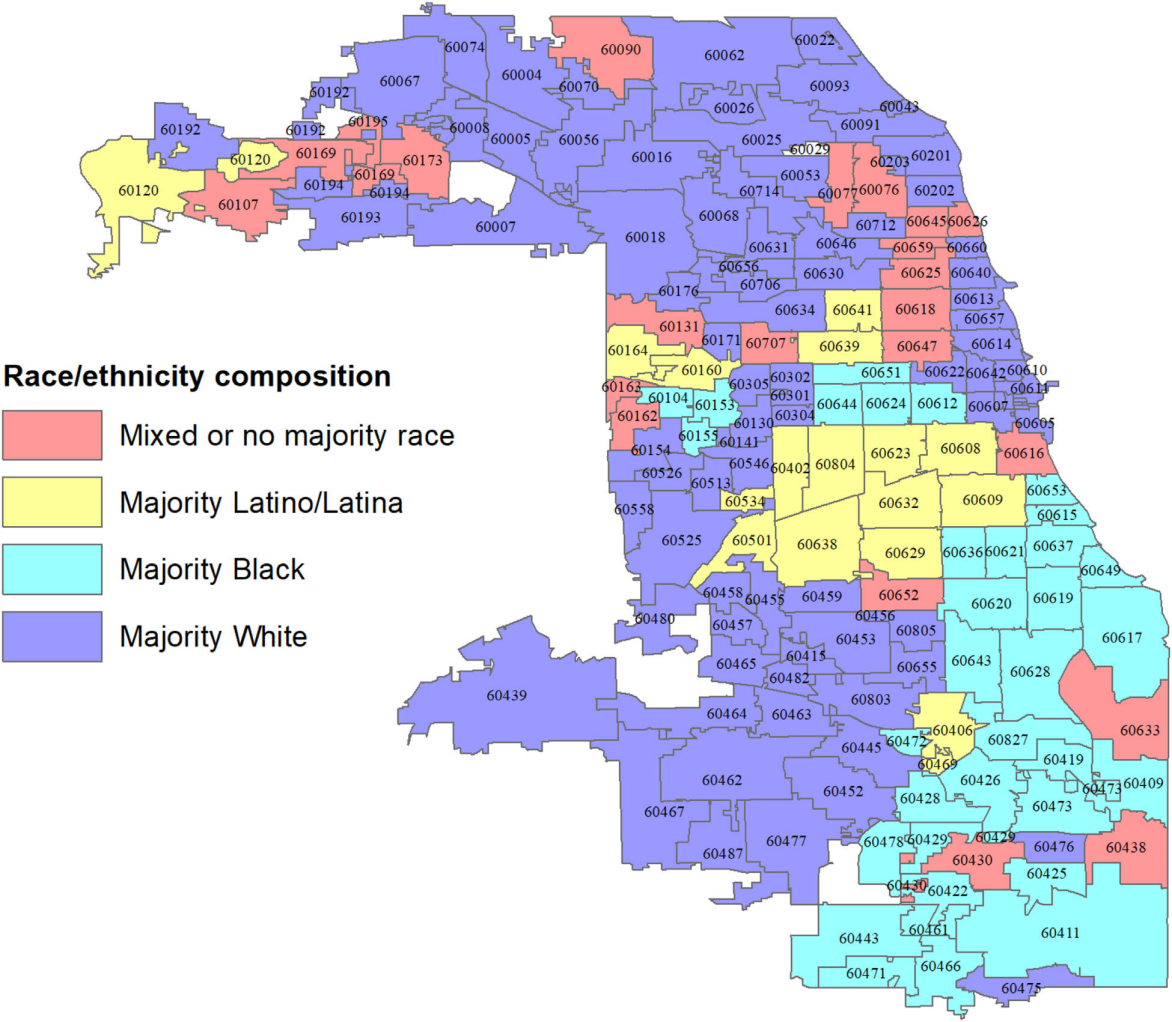
Chicago neighborhood zip code boundaries characterized by majority race/ethnicity residence.

Figure 1 is the map of the city of Chicago that displays the boundaries of all 172 zip code areas. The zip code areas are colored to depict their racial and ethnic compositions. As can be seen, neighborhoods predominantly appear to be well segregated by race and ethnicity. Most African Americans are primarily found in the southeastern section of the city. Likewise, with a few exceptions, Latinos and Latinas predominantly reside in the city's midsection neighborhoods. The rest of the communities are White‐majority neighborhoods, with mixed‐race and ethnic neighborhoods scattered throughout the city.

### Study variables and data sources

The study utilized secondary data from a public reporting site to provide insight into whether there is significant variability in COVID‐19 outcomes among minority populations that reside in a segregated city neighborhood. We used zip code areas as the basic neighborhood units and summarized the spread of the disease between March 2, 2020, and December 31, 2020, within these neighborhood units. We focused on three aspects of the spread patterns of COVID‐19—testing, positivity, and case fatality.

We defined neighborhoods with concentrations of three racial and ethnic groups—White, Black or African American, and Latino/Latina. If a particular residence zip code consists of 50% or more of each of these three race/ethnic groups, we consider that residence zip code as a concentrated neighborhood by the given race or ethnic group. For example, if the population of Whites in a particular residence zip code consists of 50% or more Whites, we consider that zip code a White (concentration) residence.

We used two data sources we linked before performing our analysis. The first data was obtained from the “Chicago Data Reports” website published by the city's coronavirus response center [15]. This dataset contained the number of individuals tested, the number of positive test results, and the number of deaths due to COVID‐19 by zip code areas within Cook County. Cook County Health's Office of Research and Regulatory Affairs approved using the data for this research.

The second data source was obtained from the US Bureau of the Census. These data provided detailed demographic and socioeconomic characteristics of each zip code within our focus area. The Census Bureau uses zip code tabulation areas (ZCTAs) to report data at the zip code level. The Bureau devises ZCTAs to solve the problem of frequently changing zip code boundaries. Because of the unchanging nature of ZCTAs, they are ideal for comparative purposes. ZCTAs are highly matched with zip codes. Therefore, we used a zip code‐ZCTA cross‐reference field as the “key” to link the data we obtained from the coronavirus response center's data reports to the Census Bureau's socioeconomic data report table.

### Data analysis

In this study, we implement spatial and statistical analysis approaches to demonstrate the patterns of differences and similarities of the effects of COVID‐19 on racial minorities. We used ESRI's Arc GIS software to create choropleth maps that provided the ethnic and racial residence patterns and a decent descriptive portrayal of the spatial patterns of COVID‐19 testing, positivity, and case fatality throughout our data collection. The number of residence zip codes within the city covered by this study is 172. Choropleth maps are used to show the geographic distribution of residence patterns and disease spread.

In addition to geographic depiction, we used descriptive statistical summary analyses of the patterns. We employed the inferential technique of one‐way analysis of variance (one‐way ANOVA) to perform our central research hypothesis. The main research hypotheses were that there are distinct and statistically significant racial and ethnic disparities among the various racial and ethnic groups regarding the COVID‐19 measures used by this research. The one‐way ANOVA test allows testing the research hypotheses about the existence or nonexistence of differences between the average standards (i.e., testing, positivity, and case fatality) of the ethnic/race‐based groupings of the neighborhoods. Whenever the one‐way ANOVA test detects a difference, we then examine the nature of the difference using a “post hoc” hypothesis test that allows us to perform multiple comparisons of the group averages. The post hoc comparison test we employed for this study is Tukey's honest statistical difference. All statistical significance tests use the critical alpha value of 0.05 as a criterion for rejecting or not rejecting the null hypothesis. We performed data manipulation and statistical analysis using the SAS statistical software.

### Ethical considerations

Cook County Health's Office of Research and Regulatory Services reviewed and approved the study protocol.

## RESULTS

### Spatial analysis

Out of a total of 172 Chicago zip codes, 98 neighborhood zip codes are categorized as “White majority” neighborhoods. This is because it was determined that 50% or more of the resident population of these neighborhood zip codes are Caucasians. By the same determination, 34 neighborhood zip codes were classified as Black or African American neighborhoods, and 18 were categorized as Latino/Latina neighborhoods. Twenty‐two neighborhood zip codes did not have a majority race or ethnic group residents. They are therefore classified as “mixed” or “no racial majority” neighborhoods. Figure 1 depicts the zip code neighborhood locations.

**FIGURE 2:**
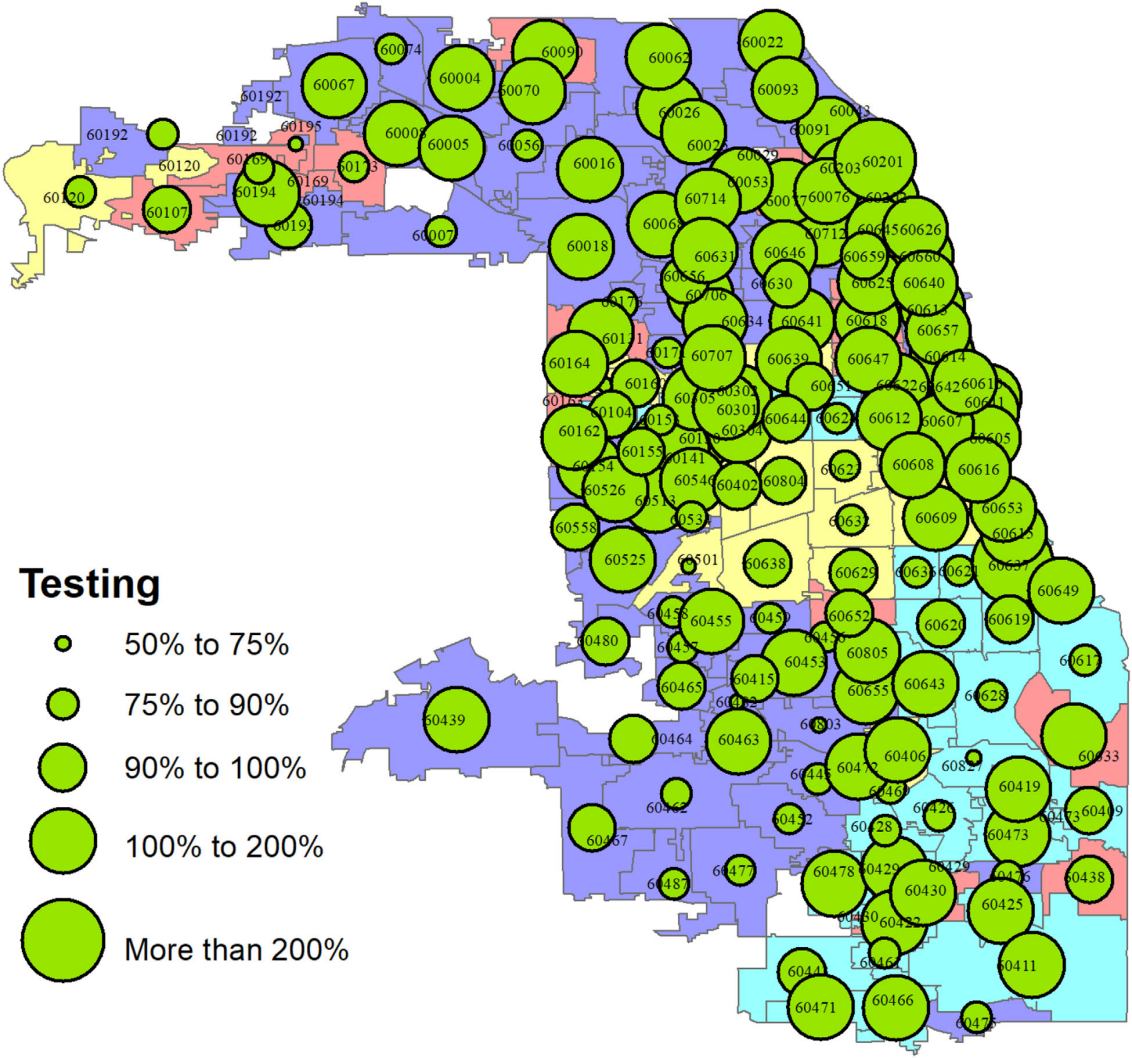
The proportion of Chicago residents who were tested for COVID‐19 by zip code: March 2 to December 31, 2020.

The following three maps visually display the geographic distribution of the COVID‐19 testing rate, positivity rate, and case fatality rate categorized by the zip codes that are the basic units of our analysis. We implement an overlay map to portray the overlap between the racial and ethnic patterns of the residents and the COVID‐19‐related events. Figure 2 shows testing rates.

As can be seen from Figure 2, testing reached all neighborhood zip codes in Chicago. The distribution portrayed on the map indicates that each neighborhood had at least 50% of its resident populations tested for COVID‐19. The average testing rate for the communities is over 100%—which means typically, a resident in a community could have received at least one test over the study period—with a few members receiving more than one test.

There are also several neighborhoods in which single individuals have received multiple tests. Neighborhood variability in the testing rates does exist, which shows testing gaps. However, these gaps do not appear to show in neighborhoods where all three race groups reside and, as such, may not indicate a racial/ethnic disparity.

**FIGURE 3:**
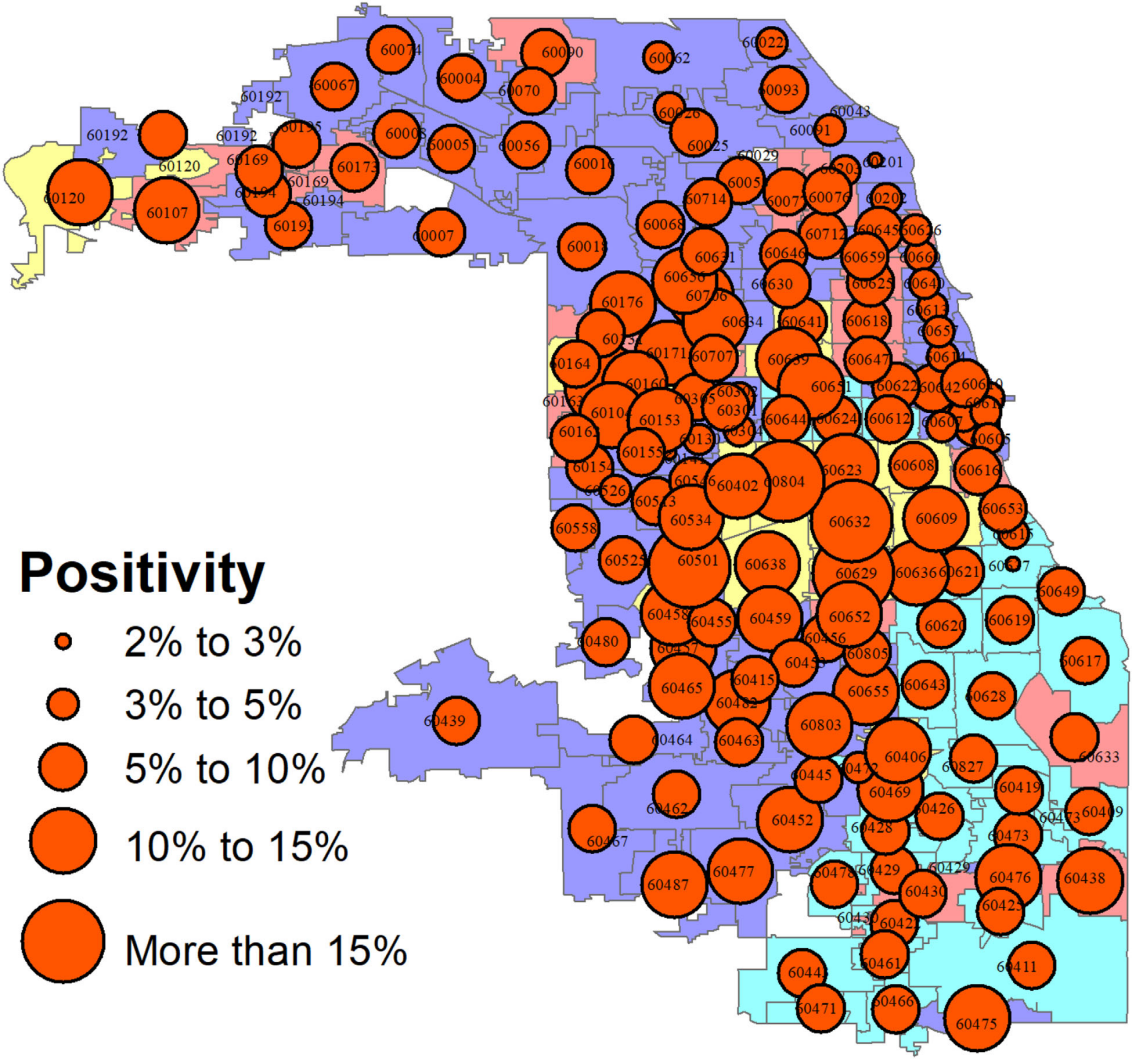
Positivity rate among individuals administered the COVID‐19 test in Chicago neighborhoods: March 2 to December 31, 2020.

Figure 3 shows the distribution of positive results among individuals who received the COVID‐19 test. The minimum positivity rate for a neighborhood is 2%, and the maximum is about 18%. The typical (average) positivity rate was about 8%. The proportional distribution of positivity tests appears to be approximately normal around this average. The geographic map of positivity seems to show a spatial pattern—such that neighborhoods in the city's midsection experienced a heavier positivity rate than those in the city's periphery.

Of particular note is that the zip codes with the highest positivity rate (i.e., greater than 15% positivity rate) are found in the city's midsection. These zip codes were previously identified as majority Latino/Latina neighborhoods. The neighborhoods with below‐average positivity rates were mainly in the city's northern end.

**FIGURE 4:**
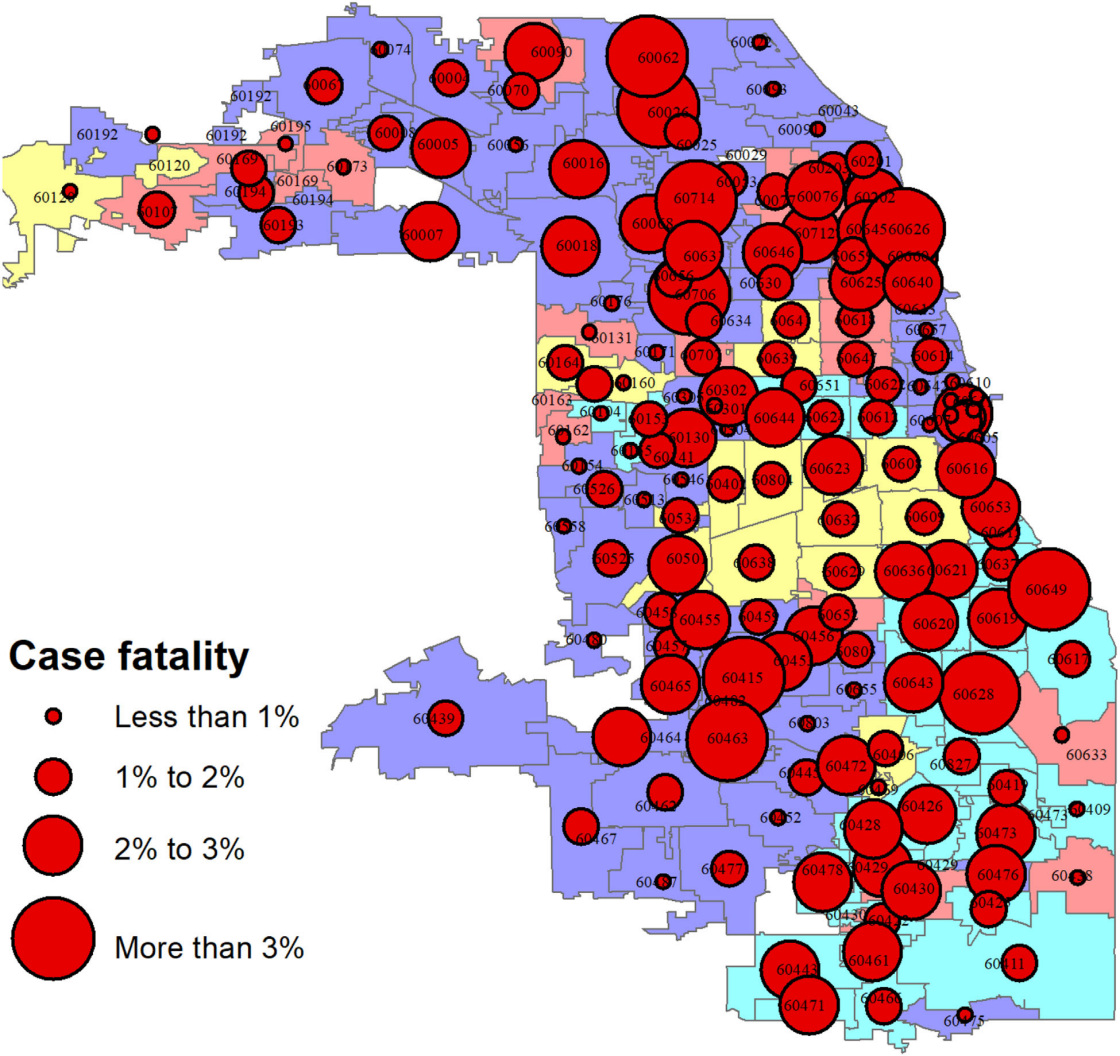
Case fatality rate or mortality rates among individuals who tested positive for COVID‐19 in Chicago neighborhoods: March 2 to December 31, 2020.

Figure 4 shows the spatial profile of fatalities among individuals who tested positive (i.e., case fatality rates) by neighborhood. The fatality rates range from less than 1% of the individuals who tested positive (0.2%, to be precise) to over 3% (6%, to be exact). The typical or average fatality rate for the neighborhoods is 1.7%, indicating a highly skewed distribution. The skew direction is positive (i.e., right‐skew)—with a few communities having extremely high‐case fatality rates. Although there is obvious spatial clustering of the high‐case fatality neighborhoods, it is impossible to discern from the map whether the clustering follows racial or ethnic neighborhood lines.

### Statistical analysis

The following analytic task was to determine if the three COVID‐19 events (i.e., testing, positivity, and case fatality) follow the race and ethnic patterns as defined by our majority race/ethnic residence system of categorizations. Such determination requires the implementation of an inferential statistical technique. As described in the Methods section, we used the one‐way ANOVA technique to perform this inferential task.

The descriptive statistics presented in Table 1 are covered in the section where we discussed the spatial distribution patterns. The summaries are presented here to provide context for the following inferential analysis and require no further description. The one‐way ANOVA partitions the overall variance of the distribution of these measures and partitions them into within group, between the group, and total variability. This is presented in Table 2, which also reports the result of the *F* test for the significance between the groups.

**TABLE 1:** Average testing, positivity, and case fatality rates of COVID‐19 among Chicago neighborhoods: March 2 to December 2020.

Residence type	Testing rate average—mean (SD)	Positivity rate average—mean (SD)	Case fatality rate average—mean (SD)
Mixed or no majority (*n* = 22)	116.76% (32.11)	8.26% (2.09)	1.66% (0.96)
Majority Hispanic (*n* = 18)	96.42% (16.47)	13.07% (2.68)	1.38% (0.50)
Majority Black (*n* = 34)	110.97% (30.06)	7.47% (2.20)	2.22% (0.96)
Majority White (*n* = 98)	130.24% (123.39)	7.25% (2.57)	1.58% (1.17)
All zip codes (*n* = 172)	121.17% (95.39)	8.03% (3.01)	1.7% (1.08)

**TABLE 2:** Analysis of variance (ANOVA) table for testing, positivity, and case fatality rates of COVID‐19 among Chicago neighborhoods.

		Sum of squares	df	Mean square	*F*	Sig.
Percent population tested	Between groups	2.305	3	0.768	0.842	0.473
	Within groups	153.301	168	0.913		
	Total	155.607	171			
Test positivity rate	Between groups	0.053	3	0.018	29.204	0.000
	Within groups	0.101	168	0.001		
	Total	0.154	171			
Case fatality rate	Between groups	0.001	3	0.000	3.788	0.012
	Within groups	0.019	168	0.000		
	Total	0.020	171			

The ANOVA reports in Table 2 describe the result of the statistical test for the difference between the average measures among the racial and ethnic categorizations of Chicago neighborhoods. The first report depicts the test for the difference in the population tested. As can be seen from the table, the differences, as given by the *F* test result, are not statistically significant (*F* = 0.84; df = 3, 168; *p* = 0.473). Based on this result, we can conclude that there is no statistically significant difference among the neighborhood groups in testing. On average, testing was administered homogeneously across the neighborhood groups. The result of the ANOVA test regarding the positivity of the COVID‐19 test, on the other hand, shows that there is a statistically significant difference between at least one race/ethnic neighborhood grouping and at least one other neighborhood grouping (*F* = 29.2; df = 3, 168; *p* < 0.001).

The multiple‐comparison post hoc tests that were conducted following the significant global ANOVA test showed that, although the neighborhoods with a majority White, majority Black, or African American and mixed‐race neighborhoods had statistically equivalent average positivity rates, the neighborhoods with a majority Hispanic population had a positivity rate that is statistically significantly higher than that of all the other race/ethnic neighborhoods (*p* < 0.001).

As shown in Table 2, the third and final measure that was examined using the one‐way ANOVA test was the COVID‐19 case fatality rate. The statistical hypothesis test using this measure showed a statistically significant difference between at least one race/ethnic neighborhood grouping and at least one other (*F* = 29.2; df. = 3, 168; *p* < 0.001).

The multiple‐comparison post hoc test that was conducted following the significant ANOVA test showed that, although the neighborhoods with majority White, majority Hispanic, and mixed‐race neighborhoods had statistically equivalent average positivity rates, the neighborhoods with majority Black or African American population, on the other hand, had a case fatality rate that is statistically significantly higher than that of all the other race/ethnic neighborhoods (*p* < 0.01).

## DISCUSSION

Our analyses were centered on evaluating neighborhood‐level patterns related to COVID‐19 events. In particular, it focused on investigating testing activities, the positivity of the test, and mortality due to COVID‐19 among Chicago neighborhoods—which we categorized by race and ethnicity characteristics. Among the main highlights of our findings is that testing was conducted throughout the city without a specific emphasis on an ethnic neighborhood. In terms of the positivity of the test, we discovered that communities with a majority Hispanic population had a markedly high positivity rate. In terms of mortality or, more specifically, case fatality rate, neighborhoods where the majority of African Americans resided had an appreciably higher fatality rate as compared to communities with a majority White or majority Hispanic population. Regarding positivity and case fatality rates, neighborhoods with a majority White or majority mixed‐race/ethnicity population had favorable odds—compared to communities in which a minority population constitutes the majority residents.

Our findings reinforce prior researchers’ conclusions that the pandemic more impacts minority residential areas in a city than nonminority residential areas. They also support our primary hypothesis that the health outcomes of health disparities are not the same across all members of the disadvantaged population. Our findings, which imply that the impact of health disparities is contextual, are relevant both for analytic as well as policy reasons.

The analytic relevance of our findings is that investigators should not consider health disparities as a one‐dimensional construct when they study the outcomes of a disease or an outbreak. Because the health outcomes of disparities can differ in different population subgroups, studies should be designed to reveal these specific health outcomes by targeting disadvantaged populations. This point is more clearly portrayed by reexamining the findings of one of the articles that we included in our literature review [14]. The authors of this article used an identical set of data as we utilized in our study. They, however, combined the Hispanic and African American neighborhoods to designate them as “minority” neighborhoods. By setting their analyses thus, they calculated the relative risk of testing positive for COVID‐19 in both populations as 1.02 (95% CI 0.95, 1.10). Note that this relative risk ratio is not statistically significant at the 0.05 level of significance, as indicated by the reported 95% confidence intervals. The correct conclusion is that the relative risk of testing positive for individuals in minority neighborhoods is not statistically significantly different from the rest of the neighborhoods. Further, the authors point out that “residents of minority ZCTA areas were 1.77 times as likely to die (IRR 1.77, [95% CI 1.17, 2.66]).” Note that although, in general, this agrees with our findings, the reported point estimate of the relative risk is likely reduced because of their analytical approach that combined groups that are dissimilar in terms of the case fatality rates. In other words, Unrun et al.’s results that we refer to herein exemplify what statisticians call “Simpson's paradox” [18].

We recommend that further research should be conducted to investigate if this pattern persists among communities in other parts of the country. In addition to confirming or negating the persistence of the patterns that we discovered in our study, we suggest that the existence of such specific patterns in terms of other diseases, particularly chronic diseases, should be studied and understood because patterns may indicate the fact that each ethnic/racial group carries distinct vulnerabilities for diseases in general and not just the current pandemic. Public health professionals should try to understand the sources of such distinct vulnerabilities among different population subgroups to develop effective disease control programs.

### Study Limitations

The limitations of the study primarily emanate from the fact that this study was an ecological analysis and did not use individual‐level data. The results characterize the neighborhoods (clusters of zip codes) and not necessarily the individuals in those neighborhoods. Other limitations, such as the underrepresentation of noncitizens, may also apply—which can impact group comparisons. In addition, the study would have benefited from adjusting the morbidity and mortality statistics to remove the impact of potential confounding factors. Adjusting for confounding factors such as preexisting comorbid conditions that exacerbate the effect of COVID‐19 was not possible due to the fact that such data are not publicly reported at the geographic level of analysis that we chose for our study.

## CONCLUSION

The study confirmed that COVID‐19 had discernable and distinct effects on each racial and/or ethnic community. The burden of morbidity was the heaviest among Hispanic populations. The high morbidity burden, however, did not translate into high mortality or high‐case fatality for this group of population. On the other hand, although the morbidity rate was relatively low among Blacks, the case fatality rate appeared to show that, given a positive test result, the probability of dying from COVID‐19 was the highest for Blacks. The study does not show the underlying causes of this pattern of outcome differentials. However, it is essential to point out that the differentials exist. There is a need for population health researchers and public health professionals to go beyond studying and theorizing about the existence of health disparities between minorities and nonminorities and explore the distinct patterns of the disparities among minority groups.

## AUTHOR CONTRIBUTIONS

Simon Geletta: *Conceptualization (lead); writing – original draft (lead); writing, review, and editing (equal); methodology (lead); formal analysis (lead)*. Kenneth Soyemi: *Resources, writing—review and editing*.

## CONFLICT OF INTEREST STATEMENT

The authors declare no conflicts of interest.

## FUNDING INFORMATION

This study did not receive support from any funding sources.

## ETHICS STATEMENT

Cook County Health's Office of Research and Regulations reviewed and approved the study protocol. As the study is based on analyzing geographic data (not human subjects), consent does not apply to our research.

## CONSENT FOR PUBLICATION

Individual‐level data are not used, and hence individuals are entirely unidentifiable, and there are no details on individuals reported within the manuscript. All authors agreed to the publication of the final and approved draft of the manuscript.

## Data Availability

The datasets generated and/or analyzed during the current study are available in (or could be extracted from) the Chicago Department of Public Health, COVID Data Reports, 2020: [Internet]. Available from: https://www.chicago.gov/city/en/sites/covid‐19/home/covid‐data‐reports.html.
